# Do routine hospital data accurately record comorbidity in advanced kidney disease populations? A record linkage cohort study

**DOI:** 10.1186/s12882-021-02301-5

**Published:** 2021-03-17

**Authors:** Ailish Nimmo, Retha Steenkamp, Rommel Ravanan, Dominic Taylor

**Affiliations:** 1 Richard Bright Renal Service, Southmead Hospital, Bristol, BS10 5NB UK; 2 UK Renal Registry, The Renal Association, Bristol, UK

**Keywords:** Comorbidity, Chronic kidney disease, Routine healthcare datasets, Record linkage, Secondary care

## Abstract

**Background:**

Routine healthcare datasets capturing clinical and administrative information are increasingly being used to examine health outcomes. The accuracy of such data is not clearly defined. We examine the accuracy of diagnosis recording in individuals with advanced chronic kidney disease using a routine healthcare dataset in England with comparison to information collected by trained research nurses.

**Methods:**

We linked records from the Access to Transplant and Transplant Outcome Measures study to the Hospital Episode Statistics dataset. International Classification of Diseases (ICD-10) and Office for Population Censuses and Surveys Classification of Interventions and Procedures (OPCS-4) codes were used to identify medical conditions from hospital data. The sensitivity, specificity, positive and negative predictive values were calculated for a range of diagnoses.

**Results:**

Comorbidity information was available in 96% of individuals prior to starting kidney replacement therapy. There was variation in the accuracy of individual medical conditions identified from the routine healthcare dataset. Sensitivity and positive predictive values ranged from 97.7 and 90.4% for diabetes and 82.6 and 82.9% for ischaemic heart disease to 44.2 and 28.4% for liver disease.

**Conclusions:**

Routine healthcare datasets accurately capture certain conditions in an advanced chronic kidney disease population. They have potential for use within clinical and epidemiological research studies but are unlikely to be sufficient as a single resource for identifying a full spectrum of comorbidities.

**Supplementary Information:**

The online version contains supplementary material available at 10.1186/s12882-021-02301-5.

## Introduction

Over 50% of individuals receiving kidney replacement therapy (KRT) have a comorbid medical condition in addition to their kidney disease [1]. Comorbidity is associated with increased hospitalisation [2], reduced quality of life [3], and mortality [4, 5]. It is therefore essential to adjust for comorbidity when comparing clinical outcomes, without which confounding due to differences in case-mix may bias results [6, 7]. Further, inaccurate or incomplete data may result in bias, so robust methods of collecting comorbidity information are required.

In clinical research studies, data are often extracted from clinical notes by specially trained staff. Benefits of this approach include collection of high-quality, consistent information with minimal missing data. However, this is resource-intensive and the economic implications of directly gathering information that is already routinely collected elsewhere need to be considered. Disease-specific registries, including the UK Renal Registry (UKRR) record comorbidity information through clinician reporting but with low data-completeness: the UKRR only captures comorbidity in half of individuals [1].

One way of improving the completeness of comorbidity data is through linkage to routinely collected healthcare datasets such as Hospital Episode Statistics (HES) [6]. These contain information recorded at the point of care delivery, are cheaper than direct data collection and of minimal burden to study participants and researchers. Long-term follow up of large populations across geographical areas can be efficiently captured with reduced attrition, no recall bias and the ability to adjust for residual confounding relating to the accrual of comorbidity over time [8–10]. If data are of sufficient quality, these datasets are an appropriate resource for use within clinical research.

HES records detailed information on National Health Service (NHS) funded hospital care in England and Wales to inform reimbursement of health providers [11]. HES data are increasingly used in research to identify participants and record outcomes [12–14], and the UKRR established HES linkage to supplement its comorbidity information in 2018 [15].

Although the accuracy of HES in recording individual medical conditions has been compared to various disease registries [16–18], its accuracy in people with advanced chronic kidney disease (CKD) is less well documented. Clustering of comorbidities [19] and higher hospitalisation rates [20] may lead to differences in the quality of data compared to the general population and merits further exploration.

The aim of this study was to investigate the accuracy of HES comorbidity data in a cohort of individuals with advanced CKD with reference to information collected by trained research nurses. This is to identify whether this resource can be reliably used within epidemiological and clinical research in the KRT population.

## Materials and methods

### Data sources and study population

We used data from the Access to Transplant and Transplant Outcome Measures (ATTOM) observational cohort study linked to the HES dataset. ATTOM recruited individuals aged 18 to 75 years in the United Kingdom between 2011 and 2013. Patients had started dialysis or received a kidney transplant within the preceding 90 days or were active on the deceased-donor waitlist, and entered ‘incident dialysis’, ‘incident transplant’ or ‘waitlisted’ cohorts respectively. Study methodology has been described previously [21].

Research nurses collected data on patient demographics, socioeconomic indicators, primary renal disease (PRD) and comorbidity (Supplementary table 1) at recruitment. Demographic and clinical data were collected from case notes whilst ethnicity and socioeconomic information were obtained from self-completed patient questionnaires. Research nurses underwent data collection training and received documentation with clear definitions against which to gather information. Independent data validation was performed by a senior nurse in a randomly selected 5% of cases with a concordance of over 98% for all collected variables [21].

Data from HES were available from 1st January 2006 to 31st December 2017, containing demographic and clinical information from NHS secondary care encounters. Encounters are recorded as admitted patient care (APC), outpatient (OP) or emergency department (ED) attendances.

Diagnoses and procedures from APC and OP episodes are coded using International Classification of Diseases 10th revision (ICD-10) and Office for Population Censuses and Surveys Classification of Interventions and Procedures version 4 (OPCS-4) criteria. Up to 20 diagnosis and 24 operation codes are recorded for each APC episode. Information in the primary position reflects the principal diagnosis, with subsequent positions documenting comorbidities collated by professional clinical coders [11].

Data were obtained by NHS Digital, stored at NHS Blood and Transplant, and linked to the ATTOM database by unique patient identifiers (Data Sharing Agreement Number DARS-NIC-14342-Q8W0X-v1.4). Ethical approval for ATTOM was obtained from the National Health Service Health and Social Care Research Ethics Committee (Ref: 11/EE/0120). Patients provided informed consent at ATTOM recruitment for subsequent analysis of outcomes. All data were stored in line with the United Kingdom Data Protection Act 1998 requirements. Study methodology was performed in line with the aforementioned ethical guidelines and regulations.

HES data were only available from hospitals in England, so ATTOM participants from elsewhere in the UK were excluded. From here we refer to ATTOM and HES as ‘study data’ and ‘hospital data’ respectively.

### Data completeness and healthcare utilisation

To determine the completeness of HES data, the dataset linkage rate and number of HES entries per individual were determined. Methodology on dataset linkage rate is described within Supplementary Material. As diagnosis recording is most detailed within HES APC [11, 22] only these episodes were used to extract comorbidity information (over 95% of OP episodes were coded as ‘unspecified morbidity’). The number of patients with an APC episode prior to study recruitment was calculated and number of admissions determined. Comorbidities among individuals with and without an APC episode were compared.

### Comorbidity recording

The comorbidities recorded by study nurses are shown in Supplementary table 1, alongside corresponding ICD-10 and OPCS-4 codes. Codes were identified from a systematic search of data dictionaries alongside consultation of established algorithms [23]. Comorbidities were extracted from all diagnosis and operation positions from hospital admissions between January 2006 and study recruitment. If a condition was recorded once, it was considered to persist on subsequent attendances in-keeping with established methodology [24]. The prevalence of comorbidities were calculated using the denominator of all individuals with dataset linkage and complete study comorbidity records.

To maximise their statistical power, studies need to identify conditions with an adequate sensitivity (proportion of true ‘cases’ identified), specificity (proportion of true ‘controls’ identified) and positive predictive value (PPV; proportion of identified cases that truly have the condition). A higher PPV leads to greater statistical power through low misclassification of positive cases which could ‘dilute’ any observed effect. False negatives have less impact on power for conditions with a relatively low prevalence as they join the larger control population. If the condition of interest is rare, specificity and negative predictive value (NPV) are generally high.

The study comorbidity dataset was taken to represent ‘gold standard’. The sensitivity, specificity, PPV and NPV of comorbidities derived from hospital data were calculated. Cohen’s kappa statistic was used to compare the agreement of recording between sources. Accepted values were taken to indicate poor (< 0.2), fair (0.21–0.40), moderate (0.41–0.6), substantial (0.61–0.8) and good (> 0.8) agreement [25]. The ICD-10 and OPCS-4 codes of comorbidities with a PPV below 50% were scrutinised to identify diagnoses giving false positive results. To examine whether disease prevalence associates with recording accuracy, pooled sensitivities and PPVs were calculated using a subgroup meta-analysis.

Operations preferentially generate cost codes for hospital episodes and the condition being treated by an operation could be more likely to be ‘truly’ present if requiring an intervention. A subgroup meta-analysis compared the sensitivity and PPV of conditions identified using ICD-10 criteria alone to those also derived from OPCS-4 codes. A random-effects model was used due to heterogeneity in the prevalence of comorbidities and variation in the sensitivity and PPV of comorbidities derived from hospital data reported previously [17, 18].

The renal modified Charlson score was calculated using comorbidities derived from study and hospital data (Supplementary table 2) [26]. The sensitivity, specificity, PPV and NPV of the Charlson score derived from hospital data were calculated.

### Statistical analyses

Descriptive statistics were used to report baseline characteristics with non-parametric continuous variables expressed as median [interquartile range, IQR] and categorical variables as frequency (percentage). The Chi-square test and Mann-Whitney U test were used to compare categorical and non-parametric continuous variables respectively. Results of regression analyses were presented as odds ratios with 95% confidence intervals. Statistical significance was defined as a *p*-value < 0.05. Analyses were performed using Stata 15 (Statacorp, College Station, TX).

## Results

### Data sets and study population

**Fig. 1:**
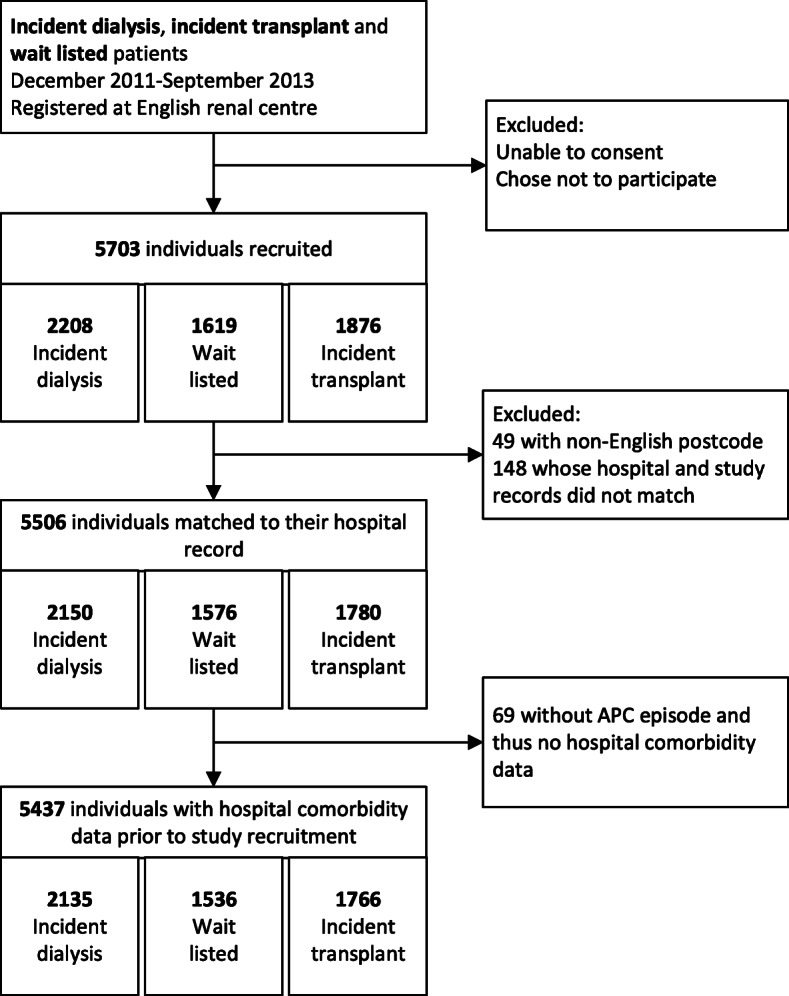
Flow chart depicting individuals included in the study. There were 69 individuals without an admitted patient care episode prior to study recruitment, but 67 of these had a subsequent admitted patient care episode after recruitment

In total, 5703 patients were recruited to ATTOM from an English renal centre. Study and hospital records were linked for 5506 (97%) individuals. Of the 197 individuals whose records did not link, 49 had non-English postcodes and likely received treatment elsewhere in the UK, leaving 148 (2.6%) unmatched (Fig. 1). Factors associated with dataset linkage are described in the Supplementary Material and shown in Supplementary table 3 and Supplementary table 4.

Of those individuals with linked datasets, the median age was 53 years [IQR 43–63], 62% of individuals were male and 76% were of white ethnicity. Overall, 20% of individuals had a PRD classified as ‘other’, with a further 19% each having diabetes and glomerulonephritis (Table 1).

**Table 1:** Study dataset linkage by patient demographic and clinical factors. Data are expressed as number (%) or median [IQR]. Standardised differences of 0.2, 0.5 and 0.8 reflect small, medium and large standardised differences respectively

Variable	Linked dataset *N* = 5506	Non-linked dataset *N* = 148	*P*	Standard diff.
Age (*n* = 5654)	53 [43–63]	51 [41–61]	0.09	0.15
Sex (*n* = 5654)				
Male	3422 (62)	84 (57)	0.18	0.11
Ethnicity (*n* = 5632)				
White	4192 (76)	100 (69)	< 0.001	0.47
Black	497 (9)	35 (24)		
Asian	750 (14)	10 (7)		
Mixed	48 (1)	0 (0)		
Index of Multiple Deprivation (n = 5654)				
1 – Most deprived	1420 (26)	31 (21)	0.51	0.11
2	1169 (21)	29 (20)		
3	1052 (19)	35 (24)		
4	983 (18)	27 (18)		
5 – Least deprived	882 (16)	26 (17)		
ATTOM cohort (*n* = 5654)				
Dialysis	2150 (39)	49 (33)	0.14	0.16
Transplant	1780 (32)	59 (40)		
Wait listed	1576 (28)	40 (27)		
PRD (*n* = 5590)				
Polycystic kidney disease	676 (13)	22 (15)	0.005	0.38
Diabetes	1026 (19)	14 (10)		
Glomerulonephritis	1057 (19)	36 (24)		
Pyelonephritis	460 (8)	15 (10)		
Hypertension	340 (6)	9 (6)		
Renovascular disease	97 (2)	7 (5)		
Other	1090 (20)	33 (22)		
Uncertain	697 (13)	11 (8)		
Charlson comorbidity index (*n* = 5571)				
0	3031 (56)	100 (68)	0.007	0.33
1–2	1518 (28)	37 (25)		
3–4	583 (11)	7 (5)		
5+	292 (5)	3 (2)		

Abbreviations: *PRD* Primary renal diagnosis

### Healthcare utilisation

The median time covered by hospital data prior to study recruitment was 6.7 years [IQR 6.4–7.0]. Of the 5506 individuals whose datasets linked, 5437 (99%) had an APC episode prior to recruitment. The median number of APC episodes was 9 [IQR 5–16] and median time from last admission to recruitment was 58 days [IQR 19–258]. Of those individuals with an admission, 89% had an admission within 1 year of recruitment and 95% within 2 years. Details of the 69 individuals without an admission prior to study recruitment are shown in the Supplementary Material; these individuals are included in subsequent analyses and counted as having no comorbidity in hospital records.

### Comorbidity recording

**Fig. 2:**
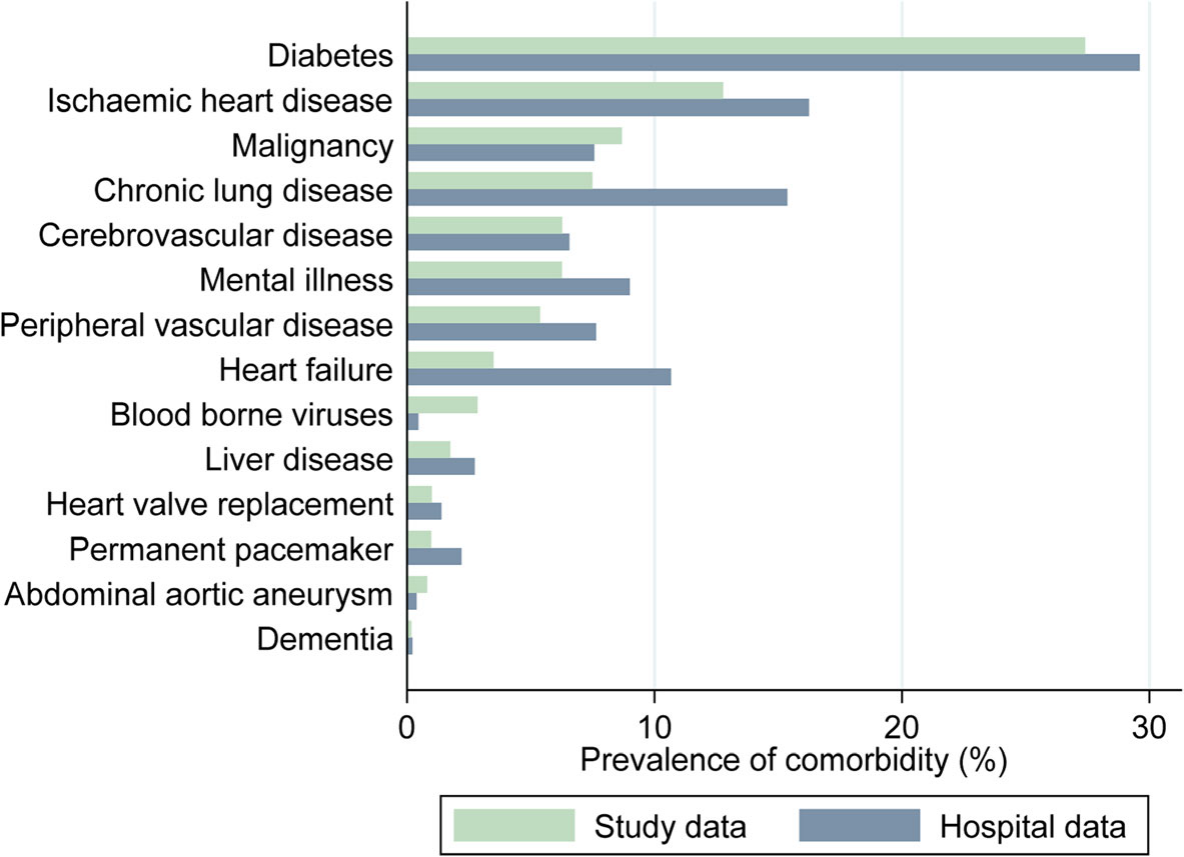
Prevalence of comorbidities derived from study and hospital datasets

**Fig. 3:**
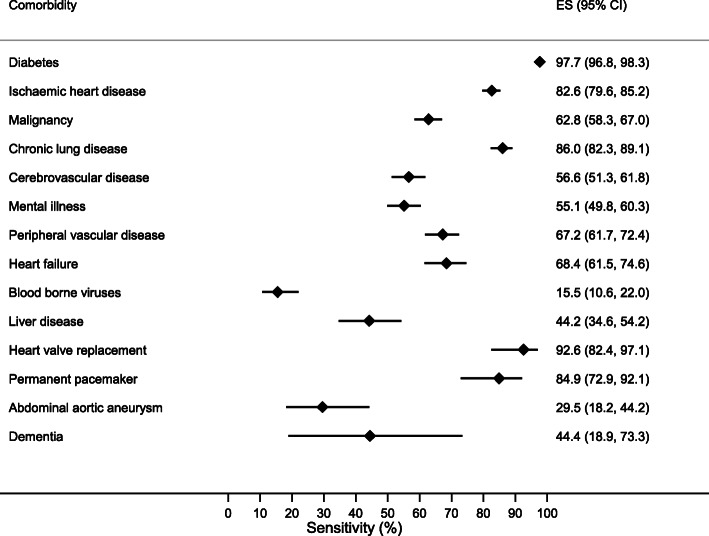
Forest plot displaying sensitivity (%) with 95% confidence intervals for individual comorbidities derived from hospital data. Comorbidities are ordered by prevalence. ES: effect size, represents sensitivity (%)

**Fig. 4:**
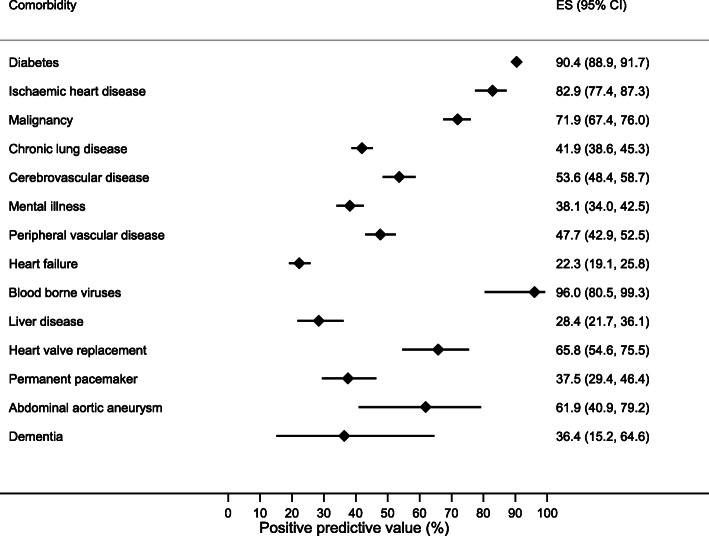
Forest plot displaying positive predictive values (%) with 95% confidence intervals for individual comorbidities derived from hospital data. Comorbidities are ordered by prevalence. ES: effect size, represents positive predictive value (%)

There was variation in the sensitivity, specificity, PPV and NPVs of comorbidities (Table 2). Diabetes, ischaemic heart disease and malignancy were most prevalent (Fig. 2) and recorded with a high sensitivity and PPV of 97.7 and 90.4% for diabetes, 82.6 and 82.9% for ischaemic heart disease and 62.8 and 71.9% for malignancy (Figs. 3 and 4). Alongside heart valve replacement, these conditions had a kappa statistic over 0.6 indicating adequate agreement.

**Table 2:** Sensitivity, specificity, positive and negative predictive values and Kappa statistic of hospital data comorbidity as compared to study data. Conditions are ordered by prevalence

Comorbidity	Sensitivity (95% CI)	Specificity (95% CI)	PPV (%) (95% CI)	NPV (%) (95% CI)	Kappa
Diabetes (*n* = 5461)	97.7 (96.8–98.4)	96.1 (95.4–96.7)	90.4 (88.9–91.8)	99.1 (98.7–99.4)	0.91
Ischaemic heart disease (*n* = 5450)	82.6 (79.6–85.4)	93.4 (92.7–94.1)	82.9 (77.3–87.6)	90.2 (89.4–91.0)	0.68
Malignancy (*n* = 5453)	62.8 (58.3–67.2)	97.7 (97.2–98.1)	71.9 (67.3–76.2)	96.5 (96.0–97.0)	0.64
Chronic lung disease (*n* = 5450)	86.0 (82.3–89.2)	90.4 (89.5–91.2)	41.9 (38.6–45.4)	98.8 (98.4–99.1)	0.52
Cerebrovascular disease (*n* = 5448)	56.6 (51.2–61.9)	96.7 (96.2–97.2)	53.6 (48.3–58.9)	97.1 (96.6–97.5)	0.52
Mental illness (*n* = 5451)	55.1 (49.7–60.5)	94.0 (93.3–94.7)	38.1 (33.8–42.6)	96.9 (96.4–97.5)	0.41
Peripheral vascular disease (*n* = 5452)	67.2 (61.5–72.6)	95.8 (95.2–96.3)	47.7 (42.8–52.6)	98.1 (97.7–98.5)	0.53
Heart failure (*n* = 5450)	68.4 (61.3–75.0)	91.4 (90.6–92.1)	22.3 (18.9–25.9)	98.8 (98.4–99.1)	0.30
Blood borne viruses (n = 5450)	15.5 (10.2–22.2)	100 (99.9–100)	96.0 (79.6–99.9)	97.6 (97.1–98.0)	0.26
Liver disease (*n* = 5452)	44.2 (34.0–54.8)	98.0 (97.6–98.4)	28.4 (21.3–36.4)	99.0 (98.7–99.3)	0.33
Heart valve replacement (*n* = 5448)	92.6 (82.1–97.9)	99.5 (99.3–99.7)	65.8 (54.0–76.3)	99.9 (99.8–100)	0.77
Permanent pacemaker (*n* = 5449)	84.9 (72.4–93.3)	98.6 (98.3–98.9)	37.5 (28.8–46.8)	99.8 (99.7–99.9)	0.51
Abdominal aortic aneurysm (*n* = 5447)	29.5 (16.8–45.2)	99.9 (99.7–99.9)	61.9 (38.4–81.9)	99.4 (99.2–99.6)	0.40
Dementia (*n* = 5453)	44.4 (13.7–78.8)	99.9 (99.7–99.9)	36.4 (10.9–69.2)	99.9 (99.8–100)	0.40

Abbreviations: *PPV* Positive predictive value, *NPV* Negative predictive value

Heart failure, chronic lung disease, mental illness and peripheral vascular disease each had greater sensitivities relative to their PPV, reflecting a greater proportion of false positive cases in hospital data. False positive cases of chronic lung disease reflected recordings of asthma or COPD in 85% of cases, and false positive cases of mental illness were recorded as depression in 46% and harmful or dependent use of alcohol in 32% of cases (Supplementary table 5). Peripheral vascular disease was identified using both ICD-10 and OPCS-4 codes and had a sensitivity of 67.2% and PPV of 47.7%. Examining the ICD-10 code alone gave a similar sensitivity (51.2, 95% CI 45.3–57.1) and PPV (51.5, 95% CI 45.6–57.4).

Blood borne viruses and abdominal aortic aneurysm had the lowest sensitivities but proportionately greater PPVs reflecting a higher rate of false negative cases. Liver disease and dementia both had poor sensitivities and PPVs under 50%. False positive liver disease cases were due to coding of liver transplant, fatty change of the liver and liver failure otherwise unspecified.

To examine whether disease prevalence was associated with the accuracy of comorbidity recording, pooled sensitivities and PPVs were calculated. The three most prevalent comorbidities comprising diabetes, heart disease and malignancy had a greater pooled PPV than all other conditions combined at 81.8% (95% CI 70.1–93.6) versus 48.1% (95% CI 37.1–59.0) (*p* < 0.001) but the association between recording accuracy and disease prevalence was not linear.

The conditions identified through ICD-10 codes alone or a combination of ICD-10 and OPCS-4 codes are shown in Supplementary table 1. There was no variation in sensitivity or PPV with coding system. The pooled sensitivity of conditions identified from ICD-10 and OPCS-4 criteria was 69.6% (95% CI 56.4–82.8), and from ICD-10 codes alone 59.8% (95% CI 39.7–80.0) (*p* = 0.43). The pooled PPV of ICD-10 and OPCS-4 diagnoses was 58.1% (95% CI 43.3–73.0) and for ICD-10 diagnoses alone was 53.5% (95% CI 29.5–77.5) (*p* = 0.74).

The sensitivity and PPV of Charlson comorbidity scores derived from hospital data are shown in Table 3. These declined with rising Charlson score. The sensitivity and PPV of a Charlson score of 0 were 88.2 and 82.9% respectively, and for a Charlson score of 1–2 were 83.9 and 66.6%.

**Table 3:** Sensitivity, specificity, positive and negative predictive values and Kappa statistic of hospital data Charlson comorbidity index as compared to study data

Charlson comorbidity index	Sensitivity (95% CI)	Specificity (95% CI)	PPV (%) (95% CI)	NPV (%) (95% CI)	Kappa
0 (*n* = 3031)	88.2 (86.8–89.5)	87.2 (86.1–88.4)	82.9 (81.3–84.4)	91.3 (90.3–92.3)	0.74
1–2 (*n* = 1518)	83.9 (82.3–85.4)	70.9 (69.3–72.5)	66.6 (64.8–68.3)	86.5 (85.1–87.7)	0.53
3–4 (*n* = 583)	73.1 (69.6–76.5)	84.7 (83.6–85.7)	39.3 (36.6–42.1)	95.9 (95.2–96.4)	0.42
5+ (*n* = 292)	67.9 (61.9–73.5)	93.0 (92.2–93.6)	32.8 (28.9–36.9)	98.3 (97.9–98.6)	0.40

Abbreviations: *PPV* Positive predictive value, *NPV* Negative predictive value

## Discussion

This observational study of over 5000 individuals with advanced CKD describes the accuracy of comorbidity recording in the Hospital Episode Statistics dataset compared to data collected by trained research nurses. The record linkage rate and proportion of individuals with comorbidity data before starting kidney replacement therapy are high, but there is variation in the sensitivity and positive predictive values of conditions derived from the hospital dataset. We suggest hospital data are adequate for capturing comorbidities including diabetes, ischaemic heart disease and malignancy but caution should be used if using this resource to identify a full spectrum of conditions.

There are several possible explanations for the variation in recording accuracy. First, accuracy may be influenced by the likelihood of a condition being directly implicated in hospital admission. Acute coronary syndromes and the management of malignancy are likely to require hospitalisation and were accurately recorded, whilst conditions predominantly monitored as an outpatient such as blood borne viruses and aortic aneurysms had lower sensitivities. Whilst the working diagnosis will influence the likelihood of hospital admission, this will also vary with clinician, social and geographical factors. We were not able to examine variation in recording accuracy between hospitals due to individuals having admissions across multiple sites and the small number of individuals attending certain hospitals, but inter-centre variation may also exist.

Second, variations in diagnostic criteria may lead to discrepancies in recording. For example, echocardiogram abnormalities are common in people on dialysis in the context of volume overload but there may not structural or functional cardiac dysfunction when the patient is at their dry weight [27]. Extracellular fluid overload could be misinterpreted as heart failure and recorded as such in clinical notes, but stricter diagnostic criteria were used in the study proforma. Variation may also reflect how ‘presumed’ diagnoses are recorded e.g. malignancy without histological confirmation.

Third, the granularity of ICD-10 and OPCS-4 coding systems should be considered. Amputations are coded as a procedure within hospital data but the reason for amputation is not documented. We assumed lower limb amputations related to peripheral vascular disease, though some may have traumatic, infective, or malignant aetiologies. Examining ICD-10 diagnosis codes for peripheral vascular disease alone did not substantially improve the PPV. Previous studies have suggested that severe disease is more likely to be correctly recorded [28], so it might have been expected that individuals with peripheral vascular disease requiring amputation to also have ICD-10 coding.

Previous studies have assessed the accuracy of hospital coding with reference to primary care and disease registry data, and recommended ways to maximise data quality. Herrett et al. examined the recording of acute myocardial infarction, reporting a PPV of 91.5% in hospital data with reference to a myocardial infarction registry. However, a third of cases were missed and they suggest linked datasets from more than one source can reduce biased estimates [16, 29]. Careful selection of ICD-10 codes is also important: a meta-analysis examining stroke recording found a wide variation in PPV, with the most accurate studies using stroke-specific as opposed to general cerebrovascular disease codes [17]. Finally, the PPV can be increased if diagnoses are recorded only if they correlate to the treating specialty, are in the primary diagnosis position or documented more than once [30]. These techniques will however reduce sensitivity so a balance must be found.

Lessons on improving routine healthcare data quality can also be taken from countries which successfully gather this information [31]. Denmark has a similar healthcare system to the UK and has excellent routine healthcare data which is easily accessible for research purposes. Consultants prospectively enter medical diagnoses in clinical databases that record the quality of healthcare delivered, and as these are used to assess treatment effectiveness and in research there are constant efforts to ensure the data is valid [32].

One study has previously examined the accuracy of HES comorbidity data in individuals on KRT, using UKRR comorbidity returns as their reference [6]. They reported overall ‘good’ concordance between sources, but the information was not as granular as is presented here and 50% of individuals had missing UKRR comorbidity information. HES comorbidity was however predictive of mortality and partially explained variation in outcomes between centres [6]. It is therefore possible that hospital data could minimise bias arising from comorbidity accrual in longitudinal observational studies [33, 34].

Using routine healthcare data for research purposes comes with economic and practical advantages: it is of low burden to participants and researchers, captures a large study population with high data completeness (96% in our study) and allows longitudinal follow up of individuals. Datasets used for hospital reimbursement also provide a ‘real-world’ view of hospitals care and insight into the financial impact of treatment.

Challenges however do exist. First, not all individuals are represented within hospital data and 2.6% of datasets in our study were not linked. This could be explained by individuals opting-out of record sharing between NHS Digital and third parties which results in the loss of 2% of hospital episodes [11].

Second, HES does not capture treatment in primary care, in the private sector or outside of England. The development of comorbidity is often associated with hospitalisation and nearly 90% of individuals had an admission within a year of KRT start, so for this population it seems unlikely for significant uncaptured community comorbidity accrual to have occurred. It is also not known if the absence of hospital data reflects no hospital contact or a loss to follow up. Similarly, hospital data cannot code conditions as absent, so lack of documentation does not definitively confirm absence of disease.

Third, the data inputted into HES are extracted from patient notes often completed by junior members of the medical team, with trained medical coders selecting the best aligned ICD-10 and OPCS-4 codes. The quality of the data depends on the documented information [35], experience of the coder and whether any systematic errors occur during the data collection process.

Finally, whilst cheaper than employing staff to gather patient information, the time and cost in gaining access to hospital data may be a barrier to its use. A new application for HES data costs £1030 and linking a bespoke dataset costs £2060 [36]. The time to receive data varies depending on the information required, but for this project took 2 years.

Our study has several strengths. We examine a large cohort of individuals with advanced CKD who are broadly representative of the UK KRT population [21] and report the accuracy of national hospital data with greater granularity and a lower rate of missing reference data than previous studies [37]. Our reference data collected by trained research nurses is likely to be accurate and reflects standard practice in most clinical research studies.

We acknowledge this study’s limitations. Study comorbidity was used as a gold standard, and although data validation suggested a high concordance between staff this source may still contain errors. Current HES data quality may differ from the 2006–2013 dataset used here. A rise in the number of completed coding fields in HES over time could yield greater data accuracy, but the possibility of over-diagnosis should be considered [37, 38].

In conclusion, the routinely collected HES dataset captured comorbidity information in 96% of individuals before the start of KRT, but there is variation in data accuracy. HES data were accurate for more prevalent conditions, but less suitable for recording a full complement of comorbidities. Understanding patterns of comorbidity among people with advanced kidney disease is crucial in informing policy and service planning, and in shared decision-making with patients. Our work will inform the use of routinely collected data to improve the efficiency of future research.

## Supplementary Information

**Additional file 1.**

## Data Availability

The HES dataset analysed during the current study is not publicly available and cannot be shared at a patient level as per the NHS Digital data sharing agreement. Analysis codes and ATTOM summary datasets are available from the corresponding author on reasonable request.

## Abbreviations

APC: Admitted patient care
ATTOM: Access to Transplant and Transplant Outcome Measures study
CKD: Chronic kidney disease
ED: Emergency department
HES: Hospital Episode Statistics
ICD-10: International Classification of Diseases version 10
IQR: Interquartile range
NHS: National Health Service
NPV: Negative predictive value
KRT: Kidney replacement therapy
OP: Outpatient
OPCS-4: Office for Population Censuses and Surveys Classification of Interventions and Procedures version 4
OR: Odds ratio
PPV: Positive predictive value
PRD: Primary renal disease
UKRR: UK Renal Registry

## References

[1] Rao A, Steenkamp R, Caskey F (2013). UK Renal Registry 16th annual report: chapter 5 comorbidities and current smoking status amongst patients starting Renal replacement therapy in England, Wales and Northern Ireland from 2011 to 2012. Nephron Clin Pract.

[2] McPhail SM (2016). Multimorbidity in chronic disease: impact on health care resources and costs. Risk Manag Healthc Policy.

[3] Gijsen R, Hoeymans N, Schellevis FG, Ruwaard D, Satariano WA, van den Bos GAM (2001). Causes and consequences of comorbidity: a review. J Clin Epidemiol.

[4] Khan IH, Catto GRD, MacLeod AM, Edward N, Fleming LW, Henderson IS (1993). Influence of coexisting disease on survival on renal-replacement therapy. Lancet.

[5] Liu J, Huang Z, Gilbertson DT, Foley RN, Collins AJ (2010). An improved comorbidity index for outcome analyses among dialysis patients. Kidney Int.

[6] Fotheringham J, Jacques RM, Fogarty D, Tomson CRV, El Nahas M, Campbell MJ (2013). Variation in Centre-specific survival in patients starting renal replacement therapy in England is explained by enhanced comorbidity information from hospitalization data. Nephrol Dial Transplant.

[7] Karamadoukis L, Ansell D, Foley RN, McDonald SP, Tomson CRV, Trpeski L, Caskey FJ (2009). Towards case-mix-adjusted international renal registry comparisons: how can we improve data collection practice?. Nephrol Dial Transplant.

[8] Cook JA, Collins GS (2015). The rise of big clinical databases. BJS (British Journal of Surgery).

[9] Grunau GL, Sheps S, Goldner EM, Ratner PA (2006). Specific comorbidity risk adjustment was a better predictor of 5-year acute myocardial infarction mortality than general methods. J Clin Epidemiol.

[10] Fraccaro P, Kontopantelis E, Sperrin M (2016). Predicting mortality from change-over-time in the Charlson Comorbidity Index: A retrospective cohort study in a data-intensive UK health system. Medicine.

[11] Herbert A, Wijlaars L, Zylbersztejn A, Cromwell D, Hardelid P (2017). Data resource profile: hospital episode statistics admitted patient care (HES APC). Int J Epidemiol.

[12] Chaudhry Z, Mannan F, Gibson-White A, Syed U, Majeed A, Ahmed S (2017). Research outputs of England’s Hospital Episode Statistics (HES) database: a bibliometric analysis. BMJ Health Care Inform.

[13] Sarween N, Hughes S, Evison F, Day C, Knox E, Lipkin G (2016). SO012 pregnancy outcomes in renal transplant recipients in England over 15 years. Nephrol Dial Transplant.

[14] Judge PK, Harper CHS, Storey BC, Haynes R, Wilcock MJ, Staplin N, Goldacre R, Baigent C, Collier J, Goldacre M, Landray MJ, Winearls CG, Herrington WG (2017). Biliary tract and liver complications in polycystic kidney disease. J Am Soc Nephrol.

[15] Renal Registry UK (2019). UK Renal Registry 21st annual report – data to 31/12/2017, Bristol, UK.

[16] Herrett E, Shah AD, Boggon R, Denaxas S, Smeeth L, van Staa T, Timmis A, Hemingway H (2013). Completeness and diagnostic validity of recording acute myocardial infarction events in primary care, hospital care, disease registry, and national mortality records: cohort study. BMJ..

[17] Woodfield R, Grant I (2015). UK biobank stroke outcomes group, UK biobank follow-up and outcomes working group, Sudlow CLM. Accuracy of electronic health record data for identifying stroke cases in large-scale epidemiological studies: a systematic review from the UK biobank stroke outcomes group. PLoS One.

[18] Yao RJR, Andrade JG, Deyell MW, Jackson H, McAlister FA, Hawkins NM (2019). Sensitivity, specificity, positive and negative predictive values of identifying atrial fibrillation using administrative data: a systematic review and meta-analysis. Clin Epidemiol.

[19] Schneider KM, O’Donnell BE, Dean D (2009). Prevalence of multiple chronic conditions in the United States’ Medicare population. Health Qual Life Outcomes.

[20] Iimuro S, Kaneko T, Ohashi Y (2019). Analysis of 2897 hospitalization events for patients with chronic kidney disease: results from CKD-JAC study. Clin Exp Nephrol.

[21] Oniscu GC, Ravanan R, Wu D, Gibbons A, Li B, Tomson C, Forsythe JL, Bradley C, Cairns J, Dudley C, Watson CJ, Bolton EM, Draper H, Robb M, Bradbury L, Pruthi R, Metcalfe W, Fogarty D, Roderick P, Bradley JA, ATTOM Investigators (2016). Access to transplantation and transplant outcome measures (ATTOM): study protocol of a UK wide, in-depth, prospective cohort analysis. BMJ Open.

[22] NHS Digital. Hospital Outpatient Activity, 2015/16: Primary Diagnosis. Published online December 1, 2016. Accessed December 9, 2019. https://digital.nhs.uk/data-and-information/publications/statistical/hospital-outpatient-activity/hospital-outpatient-activity-2015-16

[23] Quan H, Sundararajan V, Halfon P, Fong A, Burnand B, Luthi JC, Saunders LD, Beck CA, Feasby TE, Ghali WA (2005). Coding algorithms for defining comorbidities in ICD-9-CM and ICD-10 administrative data. Med Care.

[24] Wang CY, Baldwin L-M, Saver BG, Dobie SA, Green PK, Cai Y, Klabunde CN (2009). The contribution of longitudinal comorbidity measurements to survival analysis. Med Care.

[25] McHugh ML (2012). Interrater reliability: the kappa statistic. Biochem Med (Zagreb).

[26] Hemmelgarn BR, Manns BJ, Quan H, Ghali WA (2003). Adapting the charlson comorbidity index for use in patients with ESRD. Am J Kidney Dis.

[27] House AA, Wanner C, Sarnak MJ (2019). Heart failure in chronic kidney disease: conclusions from a kidney disease: improving global outcomes (KDIGO) controversies conference. Kidney Int.

[28] Baecklund E, Iliadou A, Askling J, Ekbom A, Backlin C, Granath F, Catrina AI, Rosenquist R, Feltelius N, Sundström C, Klareskog L (2006). Association of chronic inflammation, not its treatment, with increased lymphoma risk in rheumatoid arthritis. Arthritis Rheum.

[29] Millett ERC, Quint JK, De Stavola BL, Smeeth L, Thomas SL (2016). Improved incidence estimates from linked vs. stand-alone electronic health records. J Clin Epidemiol.

[30] Ludvigsson JF, Olén O, Bell M, Ekbom A, Montgomery SM (2008). Coeliac disease and risk of sepsis. Gut..

[31] Ludvigsson JF, Andersson E, Ekbom A, Feychting M, Kim JL, Reuterwall C, Heurgren M, Olausson PO (2011). External review and validation of the Swedish national inpatient register. BMC Public Health.

[32] Schmidt M, Schmidt SAJ, Adelborg K, Sundbøll J, Laugesen K, Ehrenstein V, Sørensen HT (2019). The Danish health care system and epidemiological research: from health care contacts to database records. Clin Epidemiol.

[33] Norris CM, Ghali WA, Knudtson ML, Naylor CD, Saunders LD (2000). Dealing with missing data in observational health care outcome analyses. J Clin Epidemiol.

[34] Sarfati D, Hill S, Purdie G, Dennett E, Blakely T (2010). How well does routine hospitalisation data capture information on comorbidity in New Zealand?. New Zealand Med J.

[35] Tang KL, Lucyk K, Quan H (2017). Coder perspectives on physician-related barriers to producing high-quality administrative data: a qualitative study. CMAJ Open.

[36] Data Access Request Service (DARS) charges from 2020/2021. Published online November 9, 2020. https://digital.nhs.uk/services/data-access-request-service-dars/data-access-request-service-dars-charges

[37] Fotheringham J, Fogarty D, Jacques R, El Nahas M, Campbell M (2012). Chapter 13 The Linkage of Incident Renal Replacement Therapy Patients in England (2002–2006) to Hospital Episodes and National Mortality Data: Improved Demography and Hospitalisation Data in Patients Undergoing Renal Replacement Therapy. Nephron Clin Pract.

[38] Burns EM, Rigby E, Mamidanna R, Bottle A, Aylin P, Ziprin P, Faiz OD (2012). Systematic review of discharge coding accuracy. J Public Health (Oxf).

