# Supplementary material for: Do routine hospital data accurately record comorbidity in advanced kidney disease populations? A record linkage cohort study
Source: BMC Nephrol. 2021 Mar 17;22:95. doi: 10.1186/s12882-021-02301-5 (PMC7968235; doi:10.1186/s12882-021-02301-5)
Supplement: Supplementary file 1 — Additional file 1. [file 12882_2021_2301_MOESM1_ESM.docx]

**Title:**

Do routine hospital data accurately record comorbidity in advanced kidney disease populations? A record linkage cohort study.

**Authors:**

Ailish Nimmo^1^; Retha Steenkamp^2^; Rommel Ravanan^1^; Dominic Taylor^1^ *

1. North Bristol National Health Service Trust, Bristol, UK
2. UK Renal Registry, The Renal Association, Bristol, UK

* RR and DMT contributed equally to this paper

**Running head:**

Routine healthcare data in a CKD population

**Correspondence:**

Ailish Nimmo. Richard Bright Renal Service, Southmead Hospital, Bristol, BS10 5NB, UK

ailish.nimmo@nhs.net

**Supplementary Material**

**Factors associated with dataset linkage**

Univariable and multivariable logistic regression analysis was used to examine factors associated with successful linkage of study and hospital records. Covariates defined *a priori* comprised age, sex, ethnicity, Index of Multiple Deprivation (an area-level marker of socioeconomic status ranging from 1: most deprived to 5: least deprived), study cohort, PRD and renal modified Charlson index (a weighted index of comorbidity burden ^1^). These variables were selected based on previous literature suggesting potential associations with data linkage. ^2^ ^3^ ^4^ Centre was not included in the multivariable model to prevent individuals from centres with a 100% linkage rate being dropped.

Standardised differences were used to compare characteristics between individuals with linked and non-linked datasets; values of 0.2, 0.5 and 0.8 reflected small, medium and large standardised differences respectively. ^5^ The multivariable model included complete cases only, present in 5509 (97%) of cases. Robust standard errors to account for potential intragroup correlations within centres were used.

Of 52 renal centres, 30 contained individuals whose data did not link. A funnel plot identified 5 centres as outliers (Supplementary Figure 1). It is important to note the renal centre at which individuals are registered does not necessarily represent the hospital which they attend. This is particularly relevant for kidney transplant recipients whose local renal centre is not a transplanting centre; the timing of transfer back to their local centre varies depending on local practice.

By univariable analysis, there was a reduced likelihood of datasets being linked for individuals of Black ethnicity (OR 0.34; 95% CI 0.23- 0.50) and a lower Charlson comorbidity index (Charlson score 0 vs. >5 OR 0.31; 95% CI 0.10- 0.98). Individuals with diabetic nephropathy were more likely to have linked datasets (OR 2.39; 95% CI 1.21- 4.69) (Supplementary Table 2). Significant variation was also observed between renal centres, though these vary in size (Supplementary Figure 1 and Supplementary Table 3). There was no association between dataset linkage and age, sex and ATTOM cohort which each had a standardised difference of under 0.2.

By multivariable analysis, after adjustment for age, sex, ATTOM cohort, PRD and Charlson comorbidity index, Black ethnicity remained associated with reduced likelihood of dataset linkage (OR 0.25; 95% CI 0.15- 0.41) (Supplementary Table 2). Variation was seen with socioeconomic status, but this was not linear. Previous studies have noted variation in data linkage rates with ethnicity, hypothesising that this may relate to errors in name structure or spelling and subsequent erroneous NHS numbers used for record linking. ^2^ ^6^ ^7^

**Individuals without admitted patient care prior to study recruitment**

There were 69 individuals whose datasets linked but who had no APC encounter prior to study recruitment. These individuals were more likely to be male (74% vs. 62%, p=0.04), in the waitlisted cohort (58% vs. 28%, p<0.001), have polycystic kidney disease (35% vs. 12%, p<0.001) and a lower comorbidity burden (modified Charlson index of 0: 83% vs. 56%, p<0.001).

The most prevalent comorbidity in this group was diabetes (n=7; 11%). As the number of individuals in this group was small and they had a low prevalence of comorbidity, they represent a minority of people with each condition. Individuals with blood borne viruses were most likely to not have had a prior hospital admission, but this occurred in just 2.6% of people with this diagnosis.

Of these 69 individuals, 67 had a hospital admission after study recruitment. The median time between recruitment and first admission of 917 days [IQR 333 – 1582]. Due to this length of time, we counted these individuals as having no comorbidity in hospital records in subsequent analyses.

**Supplementary References**

1. Hemmelgarn BR, Manns BJ, Quan H, Ghali WA. Adapting the charlson comorbidity index for use in patients with ESRD. *American Journal of Kidney Diseases*. 2003;42(1):125-132. doi:10.1016/S0272-6386(03)00415-3

2. Lariscy JT. Differential Record Linkage by Hispanic Ethnicity and Age in Linked Mortality Studies: Implications for the Epidemiologic Paradox. *J Aging Health*. 2011;23(8):1263-1284. doi:10.1177/0898264311421369

3. Bohensky MA, Jolley D, Sundararajan V, et al. Data Linkage: A powerful research tool with potential problems. *BMC Health Services Research*. 2010;10(1):346. doi:10.1186/1472-6963-10-346

4. Harron KL, Doidge JC, Knight HE, et al. A guide to evaluating linkage quality for the analysis of linked data. *International Journal of Epidemiology*. 2017;46(5):1699-1710. doi:10.1093/ije/dyx177

5. Cohen J. *Statistical Power Analysis for the Behavioral Sciences*. Academic press; 2013.

6. Karr AF, Taylor MT, West SL, et al. Comparing record linkage software programs and algorithms using real-world data. *PLOS ONE*. 2019;14(9):e0221459. doi:10.1371/journal.pone.0221459

7. Sayers A, Ben-Shlomo Y, Blom AW, Steele F. Probabilistic record linkage. *International Journal of Epidemiology*. 2016;45(3):954-964. doi:10.1093/ije/dyv322

**Supplementary Tables**

| ATTOM study comorbidity | ICD-10 Code | OPCS-4 Code |
| --- | --- | --- |
| Diabetes  (Type 1 or type 2) | E10.0-9  E11.0-9  E12.0-9  E13.0-9  E14.0-9 |  |
| Ischaemic heart disease  (Angina, non-ST elevation or ST elevation myocardial infarction, coronary angioplasty or coronary artery bypass graft) | I20.0-9  121.0-9  I22.0-9  I25.8 | K40-47, K48.3  K49-50  K63  K75 |
| Heart failure  (Congestive cardiac failure, right or left ventricular failure, left or right ventricular dysfunction on echocardiogram, ejection fraction below 30% on echocardiogram) | I11.0  I13.2  I50.0-1  I42.0  I42.5-9 |  |
| Cardiac valve replacement  (Previous valve replacement or repair) | Z95.2-4 | K25-29 |
| Permanent pacemaker  (Currently in situ) | Z95.0 | K60.1-9  K61.1-9 |
| Cerebrovascular disease  (Transient ischaemic attack, stroke, hemiplegia, cerebral haemorrhage, sub-arachnoid haemorrhage, subdural haemorrhage, carotid endarterectomy, carotid angioplasty or carotid operation) | I60.0-9  I61.0-9  I62.0-9  I63.0-9  I64  I65.0-9  I66.0-9  I67.0  I69.0-8  G45.0-9  G46.0-9  S06.5  S06.6 | L29  L31.1-2 |
| Peripheral vascular disease  (claudication; angioplasty, endarterectomy or bypass to iliac, femoral, popliteal, profunda, anterior tibial or posterior tibial artery; non-traumatic amputation to any limb) | I73.9 | L16  L20-21  L23  L25  L27.1-3, L27.6-9  L51-52  L54  L59-60  L634  X07-11 |
| Abdominal aortic aneurysm  (Monitored, radiological or surgical repair) | I71.3-6 | L18-19 |
| Respiratory Disease  (Asthma, COPD, emphysema, bronchiectasis) | J40 – J47  J60-67  J68.4  J70.1, J70.3 |  |
| Liver Disease  (Cirrhotic or non-cirrhotic of any cause, excludes cholecystitis and gallstone disease) | K70.0-9  K71.0-9  K72.0-9  K73.0-9  K74.0-9  K76.0  Z94.4 |  |
| Blood Borne Viruses  (Past or present infection with hepatitis B, hepatitis C or HIV either PCR or antibody positive) | B16.0-9  B17.1  B18.0-2  B20-24  Z21  R75 |  |
| Malignancy  (Any type excluding benign tumours) | C00-C97 |  |
| Mental Illness  (Depression, psychosis, bipolar disorder, substance abuse, deliberate self-harm, schizophrenia) | F10-F16 (excluding .0)  F17.2-F19 (excluding .0)  F20-25, F28-29  F30-F39  X60-X84 |  |
| Dementia  (Any form) | F00-F04  G30.0-9  G31.1 |  |

*Supplementary Table 1. Conditions recorded within the ATTOM dataset including advice to research nurses, and corresponding ICD-10 and OPCS-4 codes used to extract information from the HES dataset.*

| Comorbidity variables from Hemmelgarn et al. | Weight | Corresponding variable from ATTOM and HES datasets | Weight |
| --- | --- | --- | --- |
| Myocardial infarction | **2** | Unstable angina, myocardial infarction or coronary intervention | **2** |
| Congestive heart failure | **2** | Heart failure | **2** |
| Peripheral vascular disease  (includes Aortic aneurysm >6cm) | **1** | Peripheral vascular disease or aortic aneurysm repair | **1** |
| Cerebrovascular disease | **2** | Cerebrovascular disease | **2** |
| Dementia | **1** | Dementia | **1** |
| Chronic lung disease | **1** | Respiratory disease | **1** |
| Rheumatological /Connective tissue disease | **1** | Excluded | **-** |
| Peptic ulcer disease | **1** | Excluded | **-** |
| Diabetes without complications | **2** | Diabetes without diabetes as primary renal disease | **2** |
| Diabetes with complications | **1** | Diabetes as primary renal disease | **1** |
| Leukaemia | **2** | Leukaemia | **2** |
| Lymphoma (includes myeloma) | **5** | Lymphoma or myeloma | **5** |
| Moderate/severe liver disease | **2** | Liver cirrhosis | **2** |
| Metastatic cancer | **10** | Metastatic cancer | **10** |
| Maximum score | **33** | **Maximum score** | **31** |

*Supplementary Table 2. Comorbidities and weights included in the renal modified Charlson score.*

| Variable | Univariable model  Unadjusted OR  (95% CI) | P | Multivariable model  Adjusted OR  (95% CI) | P |
| --- | --- | --- | --- | --- |
| Age (years) (n=5654) | 1.01 (0.99 – 1.02) | 0.06 | 1.01 (0.99 – 1.02) | 0.43 |
| Sex (n=5654)  Female  Male | 1.00  1.25 (0.90 – 1.74) | -  0.18 | 1.00  1.18 (0.87 – 1.61) | -  0.28 |
| Ethnicity (n=5632)  White  Black  Asian | 1.00  0.34 (0.23 – 0.50)  1.79 (0.93 – 3.44) | -  **<0.001**  0.09 | 1.00  0.25 (0.15 – 0.41)  1.40 (0.73 – 2.67) | -  **<0.001**  0.31 |
| IMD (n=5654)  1 – Most deprived  2  3  4  5 – Least deprived | 1.00  0.88 (0.53 – 1.47)  0.66 (0.40 – 1.07)  0.79 (0.47 – 1.34)  0.74 (0.44 – 1.26) | -  0.63  0.09  0.39  0.27 | 1.00  0.93 (0.54 – 1.60)  0.55 (0.38 – 0.79)  0.64 (0.33 – 1.25)  0.61 (0.40 – 0.93) | -  0.80  **0.001**  0.19  **0.02** |
| ATTOM cohort (n=5654)  Dialysis  Transplant  Wait listed | 1.00  0.69 (0.47 – 1.01)  0.90 (0.59 – 1.37) | -  0.06  0.62 | 1.00  0.90 (0.58 – 1.41)  1.25 (0.74 – 2.11) | -  0.65  0.41 |
| PRD (n=5590)  Polycystic kidney disease  Diabetes  Glomerulonephritis  Pyelonephritis  Hypertension  Renovascular disease  Other  Uncertain | 1.00  2.39 (1.21- 4.69)  0.96 (0.56 – 1.64)  1.00 (0.51 – 1.95)  1.23 (0.56 – 2.70)  0.45 (0.19 – 1.08)  1.07 (0.62 – 1.86)  2.06 (0.99 – 4.29) | -  **0.01**  0.87  0.99  0.61  0.08  0.80  0.05 | 1.00  1.80 (0.78 – 4.15)  1.08 (0.58 – 2.04)  1.00 (0.44 – 2.30)  1.76 (0.80 – 3.86)  0.36 (0.11 – 1.18)  1.17 (0.59 – 2.33)  2.27 (0.99 – 5.22) | -  0.17  0.81  1.00  0.16  0.09  0.66  0.06 |
| Charlson index (n=5571)  5+  3-4  1-2  0 | 1.00  0.86 (0.22 – 3.33)  0.42 (0.13 – 1.38)  0.31 (0.10 – 0.98) | -  0.82  0.15  **0.04** | 1.00  0.91 (0.23 – 3.59)  0.51 (0.15 – 1.69)  0.46 (0.14 – 1.45) | 1.00  0.89  0.27  0.18 |

*Supplementary Table 3. Univariable and multivariable logistic regression analyses of factors associated with successful dataset linkage. Measures of effect are expressed as odds ratio (OR) with 95% confidence intervals. No odds ratio is expressed for Mixed ethnicity as datasets linked for all individuals. The multivariable analysis adjusted for all variables in the table with robust standard errors for centre. Effect size is presented for individuals with complete cases only (n=5509, 97%). Abbreviations: IMD; Index of Multiple Deprivation.*

| **Renal Centre** | **Individuals with linked datasets**  **N=5506** | **Individuals with non-linked datasets**  **N=148** |
| --- | --- | --- |
| Addenbrookes Hospital  Arrowe Park Hospital, Wirral  Barts and the London Hospital  Basildon Hospital  Broomfield Hospital, Chelmsford  Colchester  Cumberland Infirmary, Carlisle  Derriford Hospital  Doncaster Royal Infirmary  Dorset County Hospital  Freeman Hospital & Royal Victoria  Gloucester Royal Hospital  Guy's and St Thomas's Hospital  Heartlands Hospital  Hope Hospital, Salford  Hull Royal Infirmary  Ipswich Hospital  James Cook University Hospital  Kent & Canterbury Hospital  King's College Hospital  Leicester General Hospital  Lister Hospital, Stevenage  London - Royal Free  London - WLRaTC  New Cross Hospital, Wolverhampton  Norfolk & Norwich University Hospital  North Staffordshire - Stoke  Northern General Hospital  Nottingham City Hospital  Oxford Radcliffe Hospital  Queen Alexandra Hospital  Queen Elizabeth Hospital  R D & E Exeter  Royal Berkshire Hospital, Reading  Royal Cornwall Hospital  Royal Derby Hospital  Royal Infirmary Manchester  Royal Liverpool University Hospital  Royal Preston Hospital  Royal Shrewsbury Hospital  Royal Sussex County Hospital  Russells Hall Hospital  Southend  Southmead Hospital  St George's Hospital  St Helier Hospital, Carshalton  St James's University Hospital  St Lukes Hospital, Bradford  Sunderland Royal Hospital  University Hospital Aintree  Walsgrave Hospital, Coventry  York District General Hospital | 268 (98)  63 (100)  207 (95)  9 (100)  8 (89)  25 (100)  16 (100)  83 (100)  38 (100)  9 (100)  153 (98)  33 (100)  304 (95)  63 (97)  50 (98)  25 (96)  47 (100)  44 (100)  41 (87)  115 (100)  158 (100)  118 (97)  215 (96)  313 (97)  32 (100)  73 (100)  109 (98)  220 (98)  145 (99)  271 (99)  255 (98)  349 (97)  45 (98)  7 (100)  15 (94)  42 (100)  252 (97)  222 (97)  44 (92)  16 (100)  91 (96)  38 (100)  9 (90)  222 (97)  85 (91)  193 (97)  202 (99)  15 (100)  18 (100)  78 (100)  38 (93)  15 (100) | 6 (2)  0 (0)  11 (5)  0 (0)  1 (11)  0 (0)  0 (0)  0 (0)  0 (0)  0 (0)  3 (2)  0 (0)  16 (5)  2 (3)  1 (2)  1 (4)  0 (0)  0 (0)  6 (13)  0 (0)  0 (0)  4 (3)  9 (4)  10 (3)  0 (0)  0 (0)  2 (2)  5 (2)  2 (1)  4 (1)  6 (2)  9 (3)  1 (2)  0 (0)  6 (1)  0 (0)  7 (3)  7 (3)  4 (8)  0 (0)  4 (4)  0 (0)  1 (10)  8 (3)  8 (9)  5 (3)  1 (1)  0 (0)  0 (0)  0 (0)  3 (7)  0 (0) |

*Supplementary Table 4. Number (%) of individuals whose study dataset was successfully linked to hospital data at each renal centre.*

| Comorbidity | PPV (%)  (95% CI) | ICD-10 or OPCS-4 code | Corresponding diagnoses | False positive cases (%) |
| --- | --- | --- | --- | --- |
| Chronic lung disease | 41.9  (38.6 – 45.4) | J45.9  J44.9 | Asthma unspecified  COPD unspecified | 63  22 |
| Mental illness | 38.1  (33.8 – 42.6) | F32.9  F10.1  F10.2 | Depression unspecified  Harmful use of alcohol  Alcohol dependence | 46  18  14 |
| Peripheral vascular disease | 47.7  (42.8 – 52.6) | X11.8  L27.1  X10.4  X11.1 | Amputation toe, other  Endovascular stent graft for infrarenal AAA  Amputation through metatarsal bones  Amputation great toe | 13  9  8  8 |
| Heart failure | 22.3  (18.9 – 25.9) | I50.1  I50.0 | Congestive heart failure  Left ventricular failure | 47  39 |
| Liver disease | 28.4  (21.3 – 36.4) | Z94.4  K76.0  K72.9 | Liver transplant  Fatty change of liver  Hepatic failure | 30  20  19 |
| Permanent pacemaker | 37.5  (28.8 – 46.8) | K61.1  Z95.0 | Implantation of cardiac pacemaker system  Presence of electronic cardiac device | 62  24 |
| Dementia | 36.4  (10.9 – 69.2) | F03  F01.9 | Dementia unspecified  Vascular dementia | 75  25 |

*Supplementary Table 5. ICD-10 and OPCS-4 codes for conditions with a positive predictive value (PPV) of under 50%, which were recorded as a positive case within hospital data but a negative case within study data.*

**Supplementary Figure**

**

*Supplementary Figure 1. Funnel plot demonstrating proportion of individuals that linked with hospital data by renal centre with 95% and 99.8% limits. The dotted black line shows the mean value across all centres.*
